# Comprehensive and Rapid Chemical Profiling of *Cirsium japonicum* DC Utilizing UHPLC‐Q‐Orbitrap MS With Parallel Reaction Monitoring

**DOI:** 10.1155/jamc/5782099

**Published:** 2026-01-30

**Authors:** Jia-Yi Wang, Yu-Feng Zou, Kai-Lin Li, Yi-Fan Chen, Ling Liu, Shu-Sen Wu, Hui Li

**Affiliations:** ^1^ School of Pharmaceutical Sciences, Hunan University of Medicine, Huaihua, China, hnucm.edu.cn

**Keywords:** chemical composition, *Cirsium japonicum* DC, UHPLC-Q-exactive Orbitrap MS

## Abstract

*Cirsium japonicum*, a traditional medicinal plant, has been widely used for its therapeutic properties in treating various ailments. However, a comprehensive analysis of its chemical composition remains limited, hindering a full understanding of its chemical basis and pharmacological activities. This study aims to identify and characterize the chemical constituents of *C. japonicum* using ultra‐high‐performance liquid chromatography coupled with quadrupole‐Orbitrap mass spectrometry (UHPLC‐Q‐Exactive Orbitrap MS) and parallel reaction monitoring (PRM). A total of 94 compounds were identified, including 57 organic acids, 25 flavonoids, 3 phenylpropanoids, and 9 other components, with chlorogenic acid dominating the organic acid fraction. Organic acids and caffeic acid, among organic acid compounds, exhibit hemostatic effects based on prior evidence. The UHPLC‐Q‐Exactive Orbitrap MS‐based approach provided a detailed chemical profile of *C. japonicum*, which could facilitate a deeper understanding of its medicinal properties and guide future pharmacological studies. The identified compounds can be used as potential biomarkers for the standardization and quality control of medicinal products of *C. japonicum*.

## 1. Introduction

*Cirsium japonicum* DC (*C. japonicum*), a member of the Asteraceae family, boasts a rich historical background. This genus encompasses over 300 species that are widespread around the world, and some species within the *Cirsium* genus are consumed for their functional food benefits [1]. *C. japonicum* is predominantly found in China, Japan, and Korea [2]. The plant is prized for its extensive pharmacological properties, such as its ability to promote hemostasis, lower blood pressure, reduce inflammation, manage diabetes, and combat fungal infections [1–3]. Particularly noteworthy is the flavonoid content, which is celebrated for its antioxidant and antitumor capabilities. These properties have led to the integration of *C. japonicum* in treatments for certain types of cancers and tumors [4]. In Japan, the essential oils extracted from its rhizomes are also valued as ingredients in fragrances. However, there is a relative scarcity of characterization of its specific chemical constituents. To better facilitate its application in medical science and to gain a deeper understanding of the quality control and chemical basis of *C. japonicum*, it is very necessary to conduct a detailed chemical profiling of *C. japonicum*.

The chemical constituents of traditional Chinese medicine (TCM) are not only a vital source for modern drug discovery but also a prerequisite for elucidating the basis of therapeutic effects. However, the study of chemical components in TCM is a formidable task due to the potential for some constituents to have unknown structures or be difficult to qualify [5–7], as well as the diversity and complexity of natural product structures [8]. Due to the ability of UHPLC‐QE‐Orbitrap‐MS to simultaneously determine multiple active components in TCM and obtain sensitive qualitative and quantitative results, increasing attention is being paid to this technology [9–12]. It is a separation and analysis technique that employs high‐performance liquid chromatography (HPLC) as a separation tool and mass spectrometry (MS) as an identification tool. It combines the high separation capability of liquid chromatography (LC) with the structural identification function of tandem mass spectrometry (MS/MS) [13]. The structural low abundance of TCM components poses analytical challenges. To obtain fragment information for low‐abundance metabolites, the parallel reaction monitoring (PRM) technology is integrated. This technology offers high specificity due to its ability to scan fragment ions in high‐resolution mode, effectively avoiding interference from co‐eluting background ions on target metabolites. As a targeted MS technique, PRM focuses on acquiring secondary MS information from specific precursor ions. This technology excels in the analysis of complex plant samples, ensuring accurate measurements even in highly complex matrix environments [14]. Based on this technology, we proposed a systematic analytical strategy utilizing UHPLC‐QE‐ Orbitrap‐MS in PRM mode, combined with predicted compounds and diagnostic fragment ions, to achieve rapid detection and identification of chemical components in complex herbal extracts. Therefore, this study aims to utilize the powerful capabilities of UHPLC‐QE‐Orbitrap‐MS to systematically identify and characterize the chemical constituents in *C. japonicum* for the first time.

## 2. Results and Discussion

**Figure 1:**
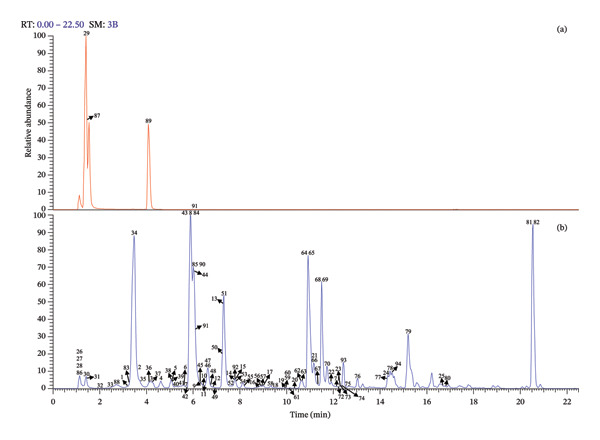
The high‐resolution extracted ion chromatogram (HREIC) in 5 ppm for multiple compounds in *C. japonicum*. (a) (in positive ion mode) m/z 130.04986, 132.1019, 205.0971, 133.06076 (b) (in negative ion mode) m/z 131.07136, 133.01424, 135.04515, 137.02441, 153.01933, 159.06628, 161.02441, 163.04006, 164.0717, 167.03498, 169.01424, 173.08193, 177.01933, 179.03498, 179.05611, 187.09758, 191.01972, 191.05611, 195.05102, 201.11323, 215.12888, 223.06119, 229.14453, 239.05611, 243.12379, 255.05102, 269.04554, 277.2173, 289.07176, 295.22786, 313.07176, 315.07215, 327.21769, 329.23334, 335.07724, 337.09289, 341.0878, 353.0878, 353.10893, 367.10345, 371.09837, 373.1504, 385.11402, 415.16097, 447.09328, 463.08819, 499.12458, 503.16175, 515.11949, 515.14062, 529.13514, 609.1461, 677.15119.

In this study, UHPLC‐QE‐Orbitrap‐MS coupled with PRM technology was utilized to identify 94 chemical constituents in *C. japonicum*, including 57 organic acids, 25 flavonoids, 3 phenylpropanoids, and 9 other types of compounds. The MS data of the detected components are shown in Table 1, the extracted ion chromatograms are presented in Figure 1, and possible fragmentation pathways are shown in Figure 2.

**Table 1:** Chromatographic and mass data of the components detected in *C. japonicum* UHPLC‐QE‐Orbitrap‐MS.

Chemical class	Peak	tR	Theoretical mass (m/z)	Experimental mass (m/z)	Error (ppm)	Formula (M)	MS/MS fragment (−)	MS/MS fragment (+)	Identification
Flavonoid	1	3.23	315.07215	315.07208	−0.24	C_13_H_16_O_9_	MS^2^ [315]: 152.0103 (100), 108.0203 (62), 153.0183 (42), 109.0283 (28)		Protocatechuic acid O‐hexoside
Flavonoid	2	3.61	341.08780	341.08728	−1.54	C_15_H_18_O_9_	MS^2^ [341]: 135.04388 (39), 161.02324 (100), 179.03391 (39)		Caffeic acid‐hexoside
Flavonoid	3	4.16	341.08780	341.08771	−0.28	C_15_H_18_O_9_	MS^2^ [341]: 161.0232 (100), 203.0340 (79), 177.0545 (31), 135.0439 (29), 179.0338 (27)		Caffeic acid‐hexoside
Flavonoid	4	4.58	341.08780	341.08743	−1.10	C_15_H_18_O_9_	MS^2^ [341]: 161.0232 (100), 179.0339 (83), 135.0438 (59)		Caffeic acid‐hexoside
Flavonoid	5	5.10	341.08780	341.08749	−0.92	C_15_H_18_O_9_	MS^2^ [341]: 161.0232 (100), 179.0339 (83), 135.0439 (39)		Caffeic acid‐hexoside
Flavonoid	6	5.61	341.08780	341.08755	−0.75	C_15_H_18_O_9_	MS^2^ [341]: 179.0339 (100), 135.0439 (37), 161.0233 (9), 136.04692 (2)		Caffeic acid‐hexoside
Flavonoid	7	5.66	289.07176	289.07153	−0.80	C_15_H_14_O_6_	MS^2^ [289]: 109.0281 (100), 125.0231 (84), 203.0702 (72), 205.0500 (48), 151.0388 (41)		Catechin^∗^
Flavonoid	8	5.89	177.01933	177.01889	−2.50	C_9_H_6_O_4_	MS^2^ [177]: 133.0283 (20), 177.0188 (14)		5,7‐Dihydroxycoumarin
Flavonoid	9	6.34	385.11402	385.11368	−0.88	C_17_H_22_O_10_	MS^2^ [385]: 164.0469 (100), 179.0704 (50), 208.0371 (48), 223.0606 (47), 149.0233 (18)		1‐O‐beta‐D‐Glucopyranosyl sinapate
Flavonoid	10	6.41	367.10345	367.10242	−2.82	C_17_H_20_O_9_	MS^2^ [367]: 191.0552 (100), 93.0332 (52), 87.0073 (17), 193.0497 (15)		3‐O‐Feruloylquinic acid
Flavonoid	11	6.51	177.01933	177.01869	−3.63	C_9_H_6_O_4_	MS^2^ [177]: 177.0182 (100), 133.0282 (18), 105.0332 (5), 149.0231 (2)		6,7‐Dihydroxycoumarin
Flavonoid	12	6.85	341.08780	341.08777	−0.10	C_15_H_18_O_9_	MS^2^ [341]: 135.0439 (37), 161.0234 (9), 179.0339 (100), 136.0474 (2)		Caffeic acid‐hexoside
Flavonoid	13	7.29	177.01933	177.01880	−3.01	C_9_H_6_O_4_	MS^2^ [177]: 177.0182 (100), 133.0282 (19), 105.0332 (5), 149.0231 (2)		Daphnetin
Flavonoid	14	7.75	373.15040	373.14996	−1.19	C_17_H_26_O_9_	MS^2^ [373]: 211.0967 (100), 59.0124 (64)		Deoxyloganin
Flavonoid	15	7.82	385.11402	385.11377	−0.65	C_17_H_22_O_10_	MS^2^ [385]: 208.0369 (100), 223.0605 (82), 164.0467 (49), 179.0703 (28), 149.0233 (8)		1‐O‐beta‐D‐Glucopyranosyl sinapate
Flavonoid	16	8.82	367.10345	367.10333	−0.34	C_17_H_20_O_9_	MS^2^ [367]: 191.05515 (100), 93.03315 (37), 173.04443 (16), 134.03615 (7), 193.04973 (12), 155.03358 (2)		4‐O‐Feruloylquinic acid
Flavonoid	17	9.01	415.16097	415.16071	−0.63	C_19_H_28_O_10_	MS^2^ [415]: 89.0230 (100), 59.0124 (38), 149.0443 (8), 131.0339 (7)		Phenylethyl primeveroside
Flavonoid	18	9.55	367.10345	367.10318	−0.75	C_17_H_20_O_9_	MS^2^ [367]: 191.05513 (100), 93.03309 (4), 134.03627 (2), 173.04434 (2), 155.03413 (1), 193.04910 (1)		5‐O‐Feruloylquinic acid
Flavonoid	19	9.95	609.14610	609.14648	0.61	C_27_H_30_O_16_	MS^2^ [609]: 300.0273 (47), 301.0338 (21), 151.0028 (4)		Quercetin 3‐O‐rhamnosyl‐galactoside
Flavonoid	20	10.35	463.08819	463.08820	0.00	C_21_H_20_O_12_	MS^2^ [463]: 300.0273 (100), 301.0344 (40), 271.0243 (11), 151.0025 (7)		Isoquercitrin^∗^
Flavonoid	21	11.19	447.09328	447.09305	−0.53	C_21_H_20_O_11_	MS^2^ [447]: 284.0323 (100), 285.0388 (31), 255.0297 (30), 227.0345 (13)		Kaempferol‐3‐O‐glucoside^∗^
Flavonoid	22	11.85	499.12458	499.12424	−0.69	C_25_H_24_O_11_	MS^2^ [499]: 191.0552 (100), 163.0390 (16), 173.0446 (13), 161.0232 (8), 93.0332 (8)		p‐Coumaroylcaffeoylquinic acids
Flavonoid	23	12.31	499.12458	499.12454	−0.09	C_25_H_24_O_11_	MS^2^ [499]: 191.0552 (72), 173.0446 (55), 59.0125 (52)		p‐Coumaroylcaffeoylquinic acids
Flavonoid	24	14.33	269.04554	269.04559	0.16	C_15_H_10_O_5_	MS^2^ [269]: 117.0333 (100), 151.0025 (38), 149.0232 (37), 107.0125 (36), 269.0453 (31)		Apigenin^∗^
Flavonoid	25	16.69	313.07176	313.07172	−0.13	C_17_H_14_O_6_	MS^2^ [313]: 298.0477 (100), 269.0452 (49), 59.0125 (15), 241.0490 (15)		Pectolinarigenin
Organic acid	26	1.16	133.01424	133.01369	−4.18	C_4_H_6_O_5_	MS^2^ [133]: 115.0023 (100), 71.0124 (46), 133.0129 (35), 72.9916 (8)		Malic acid
Organic acid	27	1.16	191.01972	191.01886	−4.53	C_6_H_8_O_7_	MS^2^ [191]: 111.0074 (16), 85.0280 (16)		Citric acid
Organic acid	28	1.16	195.05102	195.05031	−3.67	C_6_H_12_O_7_	MS^2^ [195]: 75.0073 (100), 129.0180 (64), 87.0073 (21), 99.0074 (17)		Gluconic acid
Organic acid	29	1.38	130.04986	130.04973	−1.07	C_5_H_7_NO_3_		MS^2^ [130]: 84.0447 (100), 130.0495 (21)	L‐Pyroglutamic acid
Organic acid	30	1.39	191.01972	191.01935	−1.97	C_6_H_8_O_7_	MS^2^ [191]: 111.0074 (17), 85.0281 (7)		Isocitric acid
Organic acid	31	1.44	191.05611	191.05553	−3.04	C_7_H_12_O_6_	MS^2^ [191]: 131.0337 (100), 101.0594 (37), 87.0437 (17)		Quinic acid^∗^
Organic acid	32	2.03	169.01424	169.01399	−1.52	C_7_H_6_O_5_	MS^2^ [169]: 125.0231 (100)		2,3,4‐Trihydroxybenzoic acid
Organic acid	33	2.45	371.09837	371.09818	−0.51	C_16_H_20_O_10_	MS^2^ [371]: 191.0551 (100), 173.0444 (5), 135.0442 (3)		3‐O‐Hydroxydihydro‐caffeoylquinic acid
Organic acid	34	3.44	255.05102	255.05057	−1.79	C_11_H_12_O_7_	MS^2^ [255]: 165.0546 (100), 179.0339 (31), 107.0489 (17), 149.0596 (13)		Piscidic acid
Organic acid	35	3.75	153.01933	153.01865	−4.457	C_7_H_6_O_4_	MS^2^ [153]: 109.0281 (100), 153.0181 (23), 110.0315 (4), 108.0203 (2)		Protocatechuic acid
Organic acid	36	4.12	515.14062	515.14105	0.82	C_22_H_28_O_14_	MS^2^ [515]: 191.0552 (100), 179.0340 (69), 173.0446 (44), 341.0876 (25), 353.1089 (11)		Caffeoylquinic acid‐hexoside
Organic acid	37	4.23	353.08780	353.08746	−0.98	C_16_H_18_O_9_	MS^2^ [353]: 191.0551 (100), 179.0339 (266), 135.0438 (39), 161.0235 (4), 173.0446 (3)		Neochlorogenic acid^∗^
Organic acid	38	5.07	137.02441	137.02347	−6.91	C_7_H_6_O_3_	MS^2^ [137]: 93.0334 (2), 109.0281 (2), 136.0155 (2), 108.0201 (1)		4‐Hydroxybenzoic acid
Organic acid	39	5.12	515.14062	515.14001	−1.20	C_22_H_28_O_14_	MS^2^ [515]: 173.0448 (1), 179.0338 (5), 191.0552 (100)		Caffeoylquinic acid‐hexoside
Organic acid	40	5.27	153.01933	153.01857	−4.98	C_7_H_6_O_4_	MS^2^ [153]: 109.0281 (100), 153.0181 (52), 108.0203 (34), 123.0075 (6)		Gentisuric Acid
Organic acid	41	5.44	337.09289	337.09250	−1.16	C_16_H_18_O_8_	MS^2^ [337]: 191.0552 (100), 93.0332 (42), 163.0390 (16), 173.0447 (4)		3‐Coumaroylquinic acid
Organic acid	42	5.75	515.14062	515.14026	−0.72	C_22_H_28_O_14_	MS^2^ [515]: 173.0450 (7), 179.0339 (15), 191.0551 (100)		Caffeoylquinic acid‐hexoside
Organic acid	43	5.89	353.08780	353.08728	−1.49	C_16_H_18_O_9_	MS^2^ [353]: 191.0551 (100), 173.0445 (17), 179.0338 (13), 135.0438 (10), 85.0281 (1)		Chlorogenic acid^∗^
Organic acid	44	6.06	515.14062	515.14124	1.19	C_22_H_28_O_14_	MS^2^ [515]: 191.0552 (100), 179.0340 (89), 353.0876 (24), 135.0439 (11)		Caffeoylquinic acid‐hexoside
Organic acid	45	6.32	159.06628	159.06577	−3.22	C_7_H_12_O_4_	MS^2^ [159]: 97.0645 (100), 115.0751 (34), 159.0652 (33), 95.0488 (10)		Pimelic acid
Organic acid	46	6.63	179.03498	179.03445	−2.97	C_9_H_8_O_4_	MS^2^ [179]: 135.0439 (100)		Caffeic acid^∗^
Organic acid	47	6.63	135.04515	135.04448	−4.98	C_8_H_8_O_2_	MS^2^ [135]: 135.0440 (100), 134.8642 (7)		O‐Toluic acid
Organic acid	48	6.75	515.11949	515.11920	−0.58	C_25_H_24_O_12_	MS^2^ [515]: 191.0552 (100), 179.0337 (5), 161.0233 (31), 173.0450 (2)		Dicaffeoylquinic acid
Organic acid	49	6.92	515.14062	515.14044	−0.37	C_22_H_28_O_14_	MS^2^ [515]: 173.0440 (2), 179.0337 (5), 191.0552 (100), 323.0769 (27)		Caffeoylquinic acid‐hexoside
Organic acid	50	7.24	163.04006	163.03943	−3.91	C_9_H_8_O_3_	MS^2^ [163]: 119.0490 (100), 65.0383 (3)		p‐Coumaric acid
Organic acid	51	7.31	353.08780	353.08752	−0.81	C_16_H_18_O_9_	MS^2^ [353]: 191.0551 (100), 173.0443 (5), 179.0338 (4), 135.0438 (3), 85.0280 (2)		Caffeoylquinic acid
Organic acid	52	7.58	335.07724	335.07739	0.45	C_16_H_16_O_8_	MS^2^ [335]: 135.0440 (100), 179.0341 (38)		5‐Caffeoylshikimic acids
Organic acid	53	7.84	337.09289	337.09280	−0.27	C_16_H_18_O_8_	MS^2^ [337]: 191.0552 (100), 93.0331 (24), 163.0389 (13), 173.0444 (10)		4‐Coumaroylquinic acid
Organic acid	54	8.13	515.11949	515.11908	−0.81	C_25_H_24_O_12_	MS^2^ [515]: 191.0551 (100), 179.0339 (87), 135.0439 (25), 353.0877 (2), 161.0233 (8), 173.0444 (6)		1,3‐Dicaffeoylquinic acid^∗^
Organic acid	55	8.18	335.07724	335.07721	−0.09	C_16_H_16_O_8_	MS^2^ [335]: 179.0340 (100), 135.0439 (84), 161.0233 (30), 93.0331 (11)		4‐Caffeoylshikimic acids
Organic acid	56	8.89	163.04006	163.03941	−4.03	C_9_H_8_O_3_	MS^2^ [163]: 119.0489 (100)		3‐Hydroxycinnamic acid
Organic acid	57	8.94	337.09289	337.09286	−0.09	C_16_H_18_O_8_	MS^2^ [337]: 191.0551 (100), 93.0332 (4), 163.0387 (2), 173.0443 (2),		5‐Coumaroylquinic acid
Organic acid	58	9.33	173.08193	173.08128	−3.77	C_8_H_14_O_4_	MS^2^ [173]: 111.0802 (100), 173.0808 (31), 83.0488 (7), 129.0908 (6)		Suberic acid
Organic acid	59	9.97	515.14062	515.14001	−1.20	C_22_H_28_O_14_	MS^2^ [515]: 173.0445 (100), 179.0339 (5), 191.0552 (37)		Caffeoylquinic acid‐hexoside
Organic acid	60	9.97	677.17232	677.17218	−0.21	C_31_H_34_O_17_	MS^2^ [677]: 191.0552 (100), 323.0768 (34), 179.0339 (21), 161.0232 (18), 135.0441 (3), 173.0445 (3), 335.0778 (2)		Dicaffeoylquinic acid‐hexoside
Organic acid	61	10.12	223.06119	223.06062	−2.59	C_11_H_12_O_5_	MS^2^ [223]: 208.0369 (100), 164.0467 (53), 193.0134 (47), 149.0232 (41), 152.0103 (30)		Sinapinic acid
Organic acid	62	10.62	131.07136	131.07103	−2.58	C_6_H_12_O_3_	MS^2^ [131]: 85.0644 (100), 86.0678 (3), 129.0546 (1) 113.0596 (1)		2‐Hydroxyhexanoate
Organic acid	63	10.66	677.17232	677.17157	−1.11	C_31_H_34_O_17_	MS^2^ [677]: 191.0551 (100), 323.0769 (47), 161.0232 (23), 179.0340 (23), 173.0445 (6)		Dicaffeoylquinic acid‐hexoside
Organic acid	64	10.95	515.11949	515.11877	−1.42	C_25_H_24_O_12_	MS^2^ [515]: 191.0552 (100), 179.0339 (10), 135.0439 (4), 161.0232 (3), 173.0447 (1)		1,5‐Dicaffeoylquinic acid^∗^
Organic acid	65	10.95	335.07724	335.07724	−0.00	C_16_H_16_O_8_	MS^2^ [335]: 179.0339 (100), 93.0331 (36), 135.0439 (78), 161.0232 (34),		3‐Caffeoylshikimic acids
Organic acid	66	11.19	137.02441	137.02345	−7.06	C_7_H_6_O_3_	MS^2^ [137]: 93.0332 (100)		Salicylic acid^∗^
Organic acid	67	11.35	353.10893	353.10727	−4.71	C_13_H_21_O_11_	MS^2^ [353]: 173.0445 (13), 191.0552 (9)		Quinic acid‐hexoside
Organic acid	68	11.52	187.09758	187.09691	−3.59	C_9_H_16_O_4_	MS^2^ [187]: 125.0959 (100), 187.0969 (28), 97.0645 (9), 141.8674 (1)		Azelaic acid^∗^
Organic acid	69	11.52	515.11949	515.11877	−1.42	C_25_H_24_O_12_	MS^2^ [515]: 191.0552 (100), 179.0339 (25), 135.0440 (5), 161.0235 (4), 173.0444 (26)		Dicaffeoylquinic acid
Organic acid	70	11.71	353.10893	353.10724	−4.80	C_13_H_21_O_11_	MS^2^ [353]: 191.0552 (100), 179.0339 (10) 173.1033 (2)		Quinic acid‐hexoside
Organic acid	71	12.12	677.15119	677.15088	−0.46	C_34_H_30_O_15_	MS^2^ [677]: 191.0551 (100), 179.0340 (86), 161.0233 (20), 353.0877 (15), 173.0444 (13), 335.0771 (8)		1,3,5‐Tricaffeoylquinic acid
Organic acid	72	12.21	529.13514	529.13513	−0.04	C_26_H_26_O_12_	MS^2^ [529]: 161.0233 (100), 367.1031 (28), 179.0339 (19)		Caffeoyl‐feruloylquinic acid
Organic acid	73	12.33	677.15119	677.15094	−0.37	C_34_H_30_O_15_	MS^2^ [677]: 179.0339 (100), 191.0551 (96), 173.0443 (39), 161.0233 (28), 135.0439 (23)		1,3,4‐Tricaffeoylquinic acid
Organic acid	74	12.36	515.11949	515.11908	−0.81	C_25_H_24_O_12_	MS^2^ [515]: 191.0551 (100), 179.03391 (75), 135.0439 (19), 353.0877 (3), 161.0230 (6), 173.0444 (94)		Dicaffeoylquinic acid
Organic acid	75	12.57	677.15119	677.15063	−0.83	C_34_H_30_O_15_	MS^2^ [677]: 191.0552 (100), 179.0340 (83), 135.0437 (15), 353.0874 (15)		1,4,5‐Tricaffeoylquinic acid
Organic acid	76	13.05	201.11323	201.11241	−4.09	C_10_H_18_O_4_	MS^2^ [201]: 139.1116 (100), 201.1124 (41), 183.1017 (39), 140.1148 (6), 57.0332 (4)		Decanedioic acid
Organic acid	77	14.29	229.14453	229.14395	−2.54	C_12_H_22_O_4_	MS^2^ [229]: 211.1332 (100), 229.1438 (97), 167.1430 (78), 185.1532 (3)		Dodecanedioic acid
Organic acid	78	14.35	215.12888	215.12814	−3.45	C_11_H_20_O_4_	MS^2^ [215]: 197.1174 (100), 153.1273 (91), 215.1281 (78), 125.0953 (1)		Undecanedioic acid
Organic acid	79	15.22	329.23334	329.23294	−1.24	C_18_H_34_O_5_	MS^2^ [329]: 171.1016 (100), 139.1116 (44), 99.0801 (12), 127.1117 (13)		Oleic acid
Organic acid	80	16.82	163.04006	163.03894	−6.92	C_9_H_8_O_3_	MS^2^ [163]: 119.0488 (100)		2‐Hydroxycinnamic acid
Organic acid	81	20.57	295.22786	295.22736	−1.72	C_18_H_32_O_3_	MS^2^ [295]: 277.2169 (19)		Hydroxyoctadecadienoic acid
Organic acid	82	20.57	277.21730	277.21707	−0.84	C_18_H_30_O_2_	MS^2^ [277]: 277.2168 (32), 59.0125 (14), 134.8931 (4)		Α‐Linolenic acid
Phenylpropanoids	83	3.27	167.03498	167.03455	−2.59	C_8_H_8_O_4_	MS^2^ [167]: 123.0438 (100), 151.0031 (2)		Vanillic acid
Phenylpropanoids	84	5.89	161.02441	161.02411	−1.91	C_9_H_6_O_3_	MS^2^ [161]: 133.0282 (100), 161.0233 (95), 134.0315 (6)		Umbelliferone
Phenylpropanoids	85	6.03	177.05571	177.05504	−3.83	C_10_H_10_O_3_	MS^2^ [177]: 177.0549 (100), 133.0648 (94), 162.0311 (34), 131.0492 (7)		Methyl 4‐hydroxycinnamate
Other	86	1.16	503.16175	503.16122	−1.07	C_18_H_32_O_16_	MS^2^ [503]: 89.0230 (100), 59.0124 (65), 71.0124 (50), 101, 0230 (46), 113.0231 (43)		Mannose
Other	87	1.45	132.10190	132.10156	−2.61	C_6_H_13_NO_2_		MS^2^ [132]: 86.0967 (100), 69.0704 (8), 132.1015 (3), 87.1001 (2)	L‐Leucine
Other	88	2.74	164.07170	164.07138	−1.962	C_9_H_11_NO_2_	MS^2^ [164]: 147.0439 (100), 164.0705 (45), 72.0076 (33), 148.0470 (6)		L‐Phenylalanine
Other	89	4.10	205.09715	205.09634	−3.97	C_11_H_12_N_2_O_2_		MS^2^ [205]: 146.0595 (100), 188.0699 (79), 144.0803 (16), 159.0912 (10), 118.0649 (7)	Tryptophan
Other	90	6.03	239.05611	239.05547	−2.68	C_11_H_12_O_6_	MS^2^ [239]: 179.0340 (100), 177.0546 (56), 107.0489 (32)		Eucomic acid
Other	91	6.09	133.06076	133.06032	−3.37	C_4_H_8_N_2_O_3_		MS^2^ [133]: 133.0644 (100), 105.0699 (21), 79.0547 (1)	Asparagine
Other	92	7.79	179.05611	179.05547	−3.58	C_6_H_12_O_6_	MS^2^ [179]: 59.0124 (100), 71.0124 (50), 89.0230 (34), 101.0230 (10), 119.0338 (6)		D‐Gulose
Other	93	12.45	243.12379	243.12329	−2.09	C_12_H_2_0O_5_	MS^2^ [243]: 225.1126 (100), 207.1020 (54), 99.0073 (35), 181.1226 (32), 199.1332 (25)		2‐(2‐Methylprop‐2‐enoyloxy)ethyl 6‐hydroxyhexanoate
Other	94	14.47	327.21769	327.21735	−1.06	C_18_H_32_O_5_	MS^2^ [327]: 171.1016 (100), 211.1333 (55), 229.1440 (67), 327.2173 (31), 137.0960 (13)		Corchorifatty acid F

*Note:* The numbers in parentheses of fragment ions refer to the ion abundance ratio.

^∗^For standard comparison references.

Figure 2Possible fragmentation pathways of neochlorogenic acid (a), isoquercitrin (b), caffeic acid (c), and citric acid (d).

**Figure:**
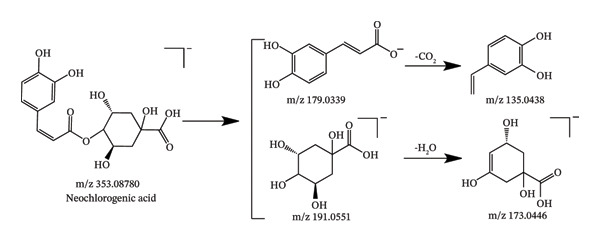
(a)

**Figure:**
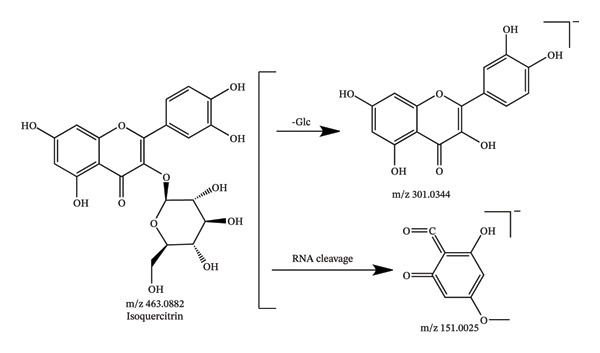
(b)

**Figure:**
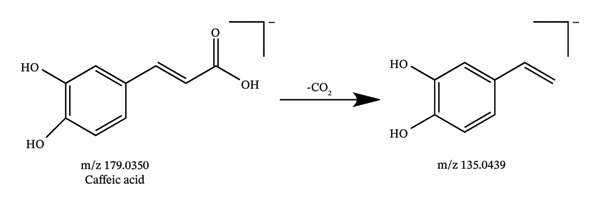
(c)

**Figure:**
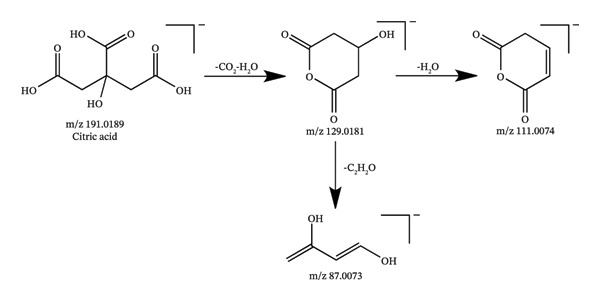
(d)

### 2.1. Analytical Strategy

To comprehensively detect and identify the chemical constituents in *C. japonicum*, a strategy based on ultra‐high‐performance liquid chromatography coupled with quadrupole‐Orbitrap mass spectrometry (UHPLC‐QE‐‐Orbitrap‐MS) and PRM was proposed. First, the *C. japonicum* sample was analyzed simultaneously in both positive and negative ionization modes using UHPLC‐QE‐Orbitrap‐MS to obtain high‐resolution MS data. Second, the data were processed using Compound Discoverer 3.0 software, combined with high‐resolution extracted ion chromatography (HREIC). Third, MS^2^ data were acquired through PRM mode for those components with trace content. Finally, 94 chemical constituents were successfully identified from *C. japonicum* based on diagnostic fragment ion information.

### 2.2. Identification of *C. japonicum*

#### 2.2.1. Identification of Flavonoid Components

Compounds 7, 20, 21, and 24, which showed the deprotonated ion [M‐H]^−^ m/z 289.0718, 463.0882, 447.0933, and 269.0455, were eluted at 5.66, 10.35, 11.19, and 14.33 min, respectively, and were accurately characterized as catechin, isoquercitrin, kaempferol‐3‐O‐glucoside, and apigenin by comparing the retention time and MS data with those of reference standards.

Compounds 8, 11, and 13 generated the same deprotonated ion [M‐H]^−^ m/z 177.0193 (C_9_H_5_O_4_) and fragment ions m/z 149.0231 [M ‐H‐CO]^−^ and 133.0282 [M ‐H‐CO_2_]^−^, suggesting they are isomers. According to the retention time, they were identified as 5,7‐dihydroxycoumarin, 6,7‐dihydroxycoumarin, and daphnetin [15].

Compound 1 was eluted at 3.23 min and possessed the quasi‐molecular ion at [M‐H]^−^ m/z 315.0722. It generated fragment ions at m/z 153.0183, which corresponds to protocatechuic acid resulting from the loss of the hexoside group as a neutral molecule, and at m/z 109.0283 due to the sequential loss of a hexose unit and subsequent loss of CO_2_ (44 Da) as a neutral species, and was identified as protocatechuic acid O‐hexoside [16]. Likewise, Compounds 14, 17, 19, and 25 were identified as deoxyloganin, phenylethyl primeveroside, quercetin 3‐O‐rhamnosyl‐galactoside, and pectolinarigenin by comparing their tR and fragments with those in the literature [11, 17, 18].

Compounds 2, 3, 4, 5, 6, and 12 were detected at 3.16, 4.16, 4.58, 5.10, 5.16, and 6.85 min, respectively. They possessed the same quasi‐molecular ion [M‐H]^−^ at m/z 341.0878 and exhibited a fragment ion at m/z 179.033 (C_9_H_7_O_4_), resulting from the neutral loss of a saccharide group with a mass of 162 Da. The base peak of m/z 135.043 (C_8_H_7_O_2_) and the fragment ion at m/z 179.033 (C_9_H_7_O_4_) were characteristic of caffeic acid‐hexoside [19].

Compounds 22 and 23 generated the same deprotonated ion [M‐H]^−^ m/z 499.12458. The appearance of fragment ions m/z 191.054 (C_7_H_11_O_6_) and 173.044 (C_7_H_9_O_5_) in the MS^2^ spectrum of those compounds were identified as p‐coumaroylcaffeoylquinic acids [19]. Compounds 10, 16, and 18, found at 6.41, 8.82, and 9.55 min, possessed the same quasi‐molecular ion at [M‐H]^−^ m/z 367.1035 and were identified as 3‐O‐feruloylquinic acid, 4‐O‐feruloylquinic acid, and 5‐O‐feruloylquinic acid by referring to the literature data [19]. Compounds 9 and 15 eluted at 6.34 and 7.82 min, respectively, and showed the same quasi‐molecular ion [M‐H]^−^ at m/z 385.1140 (C_17_H_21_O_10_). By comparison with database data, they were suggested to be 1‐O‐β‐D‐glucopyranosyl sinapate.

#### 2.2.2. Identification of Organic Acid Components

Compounds 31, 46, 66, and 68 were eluted at 1.44, 6.63, 11.19, and 11.52 min, respectively, with the deprotonated molecular ion [M‐H]^−^ at m/z 191.0561 (C_7_H_11_O_6_), 179.0350 (C_9_H_7_O_4_), 137.0244 (C_7_H_5_O_3_), and 187.0966 (C_9_H_15_O_4_), respectively; these were characterized as quinic acid, caffeic acid, salicylic acid, and azelaic acid, respectively, by comparing the retention times and MS and MS^2^ information with those of the standards. Compounds 54 and 64 were found at 8.13 and 10.95 min, respectively, possessing the quasimolecular ion [M‐H]^−^ at m/z 515.1195, and were unambiguously identified as 1,3‐dicaffeoylquinic acid and 1,5‐dicaffeoylquinic acid by comparison with reference standards. Compounds 37 and 43 were found at 4.23 and 5.89 min, possessing the quasimolecular ion [M‐H]^−^ at m/z 353.0878, mass spectrum data with these reference standards neochlorogenic acid and chlorogenic acid, respectively. Thus, they were unambiguously assigned as neochlorogenic acid and chlorogenic acid.

Compounds 48, 69, and 74 possessed the same quasi‐molecular ion and mass spectrum data as those of Compounds 54 and 64 and were identified as dicaffeoylquinic acid isomers. Compound 38 was eluted at 5.07 min, possessing the same quasi‐molecular ion [M‐H]^−^ at m/z 137.0244 and fragment ions m/z 93.033 as Compound 66, suggesting they are isomers. By comparing with tR and fragments in the Mass Spectrometry Databases, Compound 38 was identified as 4‐hydroxybenzoic acid. Likewise, Compound 51, eluted at 7.31 min, was identified as chlorogenic acid isomers and precisely characterized as caffeoylquinic acid. Compounds 50, 56, and 80, with quasi‐molecular ion at [M‐H]^−^ m/z 163.0401, were eluted at 7.24, 8.89, and 16.82 min, respectively. All these compounds yielded the fragment ions m/z 119.0480; they were identified as p‐coumaric acid, 3‐hydroxycinnamic acid, and 2‐hydroxycinnamic acid by comparing the retention time with reference compounds, respectively.

Compounds 76, 77, and 78, all sharing identical fragment ion losses with Compound 67, were identified as decanedioic acid, dodecanedioic acid, and undecanedioic acid, respectively, based on their retention times in the MS analysis.

In positive ion mode, Compound 29, eluted at 1.38 min, exhibited a quasi‐molecular ion at [M+H]^+^ m/z 130.04986, generated the main fragment ion at m/z 84.0447, and was identified as L‐pyroglutamic acid [20, 21].

In negative ion mode, Compound 26, observed at 1.16 min, possessed the quasi‐molecular ion at [M‐H]^−^ m/z 133.0142 and provided the fragment ions m/z 115.0023 by the loss of water [M‐H‐H_2_O]^−^ and m/z 71.0124. It was identified as malic acid, consistent with the literature [22]. Compound 62 was detected at 10.62 min, possessing the quasimolecular ion [M‐H]^−^ at m/z 131.0714 and the main MS/MS fragment ion at m/z 129.0546 [M‐H‐H_2_]^−^, which further produces the characteristic fragment ions at m/z 113.0596 [M‐H‐H_2_O]^−^ and 85.0644 [M‐H‐C_2_H_5_OH]^−^. Therefore, they were deduced as 2‐hydroxyhexanoate [23]. Compound 33 yielded a quasimolecular ion [M‐H]^−^ at m/z 371.0984 (C_16_H_19_O_10_), was eluted at 2.45 min, and secondary peaks at m/z 191.0551 [quinic acid‐H^+^], m/z 173.0444 [quinic acid‐H_2_O‐H^+^], and m/z 135.0442 [caffeic acid‐CO_2_‐H^+^], was identified as 3‐O‐hydroxydihydrocaffeoylquinic acid [24]. Likewise, Compounds 34, 45, and 58 yielded a quasimolecular ion [M‐H]^−^ at m/z 255.0510 (C_11_H_11_O_7_), 159.0663 (C_7_H_11_O_4_), and 173.0819 (C_8_H_13_O_4_), found at 3.44, 6.32, and 9.33 min, respectively, and were tentatively identified as piscidic acid, pimelic acid, and suberic acid.

Compound 61, possessing a deprotonated ion [M‐H]^−^ at m/z 223.0612 (C_11_H_11_O_5_), was detected at 10.12 min and generated the main fragment ions at m/z 208.0369 and 193.0134 by the sequential elimination of two methyl groups (CH_3_, 15 Da) and was identified as sinapinic acid. Additionally, the detection of decarboxylated ions at m/z 164.0467 and 149.0232 is observed [25]. Compounds 35 and 40 were eluted at 3.75 and 5.27 min, respectively, possessing the same quasi‐molecular ion [M‐H]^−^ at m/z 153.0193 and identified as protocatechuic acid and gentisuric acid according to the MS and MS/MS spectra. Compounds 28 and 32 were identified as gluconic acid and 2,3,4‐trihydroxybenzoic acid.

Compounds 27 and 30, eluted at 1.16 and 1.39 min, respectively, yielded the same parent ion [M‐H]^−^ at m/z 191.0197 (C_6_H_7_O_7_), and the typical fragment ions m/z 111.0074 [M‐H‐CO_2_‐2H_2_O]^−^ and 85.0280 were characterized as citric acid and isocitric acid, respectively [26]. Compounds 67 and 70 with the same deprotonated ion [M‐H]^−^ at m/z 353.1089 (C_13_H_20_O_11_), all of those compounds yielded fragment ions at m/z 191.054 [M‐H‐C_6_H_9_O_5_]^−^ and 173.044 [M‐H‐C_6_H_11_O_6_]^−^, which are consistent with the fragment of the quinic acid moiety. Therefore, they indicated they are quinic acid‐hexoside [27].

Compounds 52, 55, and 65 were eluted at 7.58, 8.18, and 10.95 min, respectively, with the same quasi‐molecular ion [M‐H]^−^ at m/z 335.0772 (C_16_H_15_O_8_). Losing a neutral molecule resulted in m/z 179.0340, and further losses of an H_2_O moiety and CO_2_ produced m/z 161.0233 and 135.0439, respectively. Based on their retention times, they were identified as compounds 5‐caffeoylshikimic acids, 4‐caffeoylshikimic acids, and 3‐caffeoylshikimic acids [28]. Compounds 36, 39, 42, 44, 49, and 59 yielded the same quasi‐molecular ion [M‐H]^−^ at m/z 515.1406 (C_22_H_27_O_14_) and were eluted at 4.12, 5.12, 5.75, 6.06, 6.92, and 9.97 min, respectively. All these compounds showed the fragment ions at m/z 173.044 (C_7_H_9_O_5_), 179.034 (C_9_H_7_O_4_), and 191.055 (C_7_H_11_O_6_) and were proposed as caffeoylquinic acid‐hexoside [19]. Compounds 71, 73, and 75, which generated the same quasi‐molecular ion [M‐H]^−^ at m/z 677.1512, according to the published study, were tentatively identified as 1,3,5‐tricaffeoylquinic acid, 1,3,4‐tricaffeoylquinic acid, and 1,4,5‐tricaffeoylquinic acid by analyzing the retention times of those compounds, respectively [29]. Likewise, Compounds 41, 53, and 57, which showed the same deprotonated ion [M‐H]^−^ m/z 337.0929, were eluted at 5.44, 7.84, and 8.94 and were tentatively identified as 3‐coumaroylquinic acid, 4‐coumaroylquinic acid, and 5‐coumaroylquinic acid according to the base peak and retention time [30]. Compounds 39 and 63 were tentatively characterized as dicaffeoylquinic acid‐hexoside.

#### 2.2.3. Identification of Phenylpropanoids

Compound 83 exhibited a quasi‐molecular ion [M‐H]^−^ at m/z 167.0350, found at 3.27 min, and was identified as vanillic acid, which showed fragment ions at m/z 151.0031 [M‐H‐CH_3_]^−^ and 123.0438 [M‐H‐CO_2_]^−^ [31]. By comparing the retention time and fragment ions with the reference compound, Compound 85, which generated a deprotonated molecular ion [M‐H]^−^ at m/z 177.0557 (C_10_H_9_O_3_), was unambiguously characterized as methyl 4‐hydroxycinnamate.

#### 2.2.4. Identification of Other Components

Compound 87 was found at 1.45 min, possessing the quasimolecular ion [M + H]^+^ at m/z 132.1019, generating the main fragment ion at m/z 86.0967 [M + H‐HCOOH]^−^, and the typical fragment ion m/z 69.0704 by the loss of the NH_3_ group. It was identified as L‐leucine, consistent with the literature [32]. Similarly, Compound 89 was detected as tryptophan [33].

By comparing the retention time and fragment ions with reference compounds, Compounds 88 and 94 generated a deprotonated molecular ion [M‐H]^−^ at m/z 164.0717 (C_9_H_10_NO_2_), 177.0557 (C_10_H_9_O_3_), and 327.2177 (C_18_H_31_O_5_), respectively, and were unambiguously characterized as L‐phenylalanine and corchorifatty acid F.

## 3. Materials and Methods

### 3.1. Chemicals and Reagents

*C. japonicum* was obtained from Chongqin City. The reference standard Luteoloside was purchased from Baoji Herbest Bio‐Tech Co., Ltd.; Isoquercitrin; Catechin was purchased from Chengdu Herborui Biotechnology Co., Ltd.; Caffeic acid was purchased from Aladdin Company; Salicylic acid, Quinic acid was purchased from Shanghai Yuanye Biotechnology Co., Ltd.; Chlorogenic acid, Neochlorogenic acid was purchased from Chengdu Ruifensi Biotechnology Co., Ltd.; Azelaic acid was purchased from Shandong Xiya Chemical Co., Ltd.; 1,3‐Di‐caffeoylquinic acid, 1,5‐Di‐caffeoylquinic acid was purchased from Chengdu ManSite Biotechnology Co., Ltd.; Apigenin was purchased from Chengdu HerbSubstance Co., Ltd. HPLC‐grade acetonitrile, methanol, and formic acid (FA) were purchased from Thermo Fisher Scientific (Fair Lawn, NJ, USA). All other chemicals used in this study were of analytical grade and were purchased from Beijing Chemical Works (Beijing, China). Deionized water was obtained using a Milli‐Q Gradient A 10 System (Millipore, Billerica, MA, USA).

### 3.2. Sample Preparation

*C. japonicum* was ground into powder and stored for subsequent use. Each reference standard was precisely weighted and dissolved in methanol. The dried powder of *C. japonicum* (1 g) was reflux‐extracted in 50 mL methanol for 30 min, and then the extracted solution was filtered and dried under reduced pressure to yield the brown residues. Subsequently, a total of 1 mL of methanol was re‐dissolved and centrifuged (15 min, 4°C, 10,000 rpm) to obtain the supernatant for further analysis.

### 3.3. Analytical Strategy

#### 3.3.1. Instrumentation

The analysis was performed using a Dionex Ultimate 3000 ultra‐high‐performance liquid chromatography (UHPLC) system coupled with a Q‐Exactive Orbitrap mass spectrometer (Thermo Fisher Scientific, USA). The UHPLC system was equipped with a quaternary pump (LPG‐3400SD), a vacuum degasser, an autosampler, and a thermostatted column compartment. The Q‐Exactive Orbitrap mass spectrometer was equipped with an electrospray ionization (ESI) source, which allowed for high‐resolution and high‐accuracy mass measurements.

#### 3.3.2. Chromatographic Conditions

Chromatographic separation was achieved using a Waters ACQUITY UPLC BEH C18 column (100 × 2.1 mm, 1.7 μm) maintained at 45°C. The mobile phase consisted of 0.1% aqueous FA (solvent A) and acetonitrile (solvent B). A gradient elution program was employed as follows: 95%–90% C, 0–2 min; 90%–87% C, 2–5 min; 87%–75% C, 5–9 min; 75%–65% C, 9–11 min; 65%–55% C, 11–14 min; 55%–35% C, 14–17 min; 35%–25% C, 17–20 min; 20%–26% C, 20–26 min; 95% C, 26–30 min. The autosampler was maintained at 4°C during operation, while the mobile phase flow rate was set at 0.28 mL/min with an injection volume of 2 μL. To ensure optimal separation efficiency and reproducibility, the column temperature was consistently maintained at 45°C throughout the analysis.

#### 3.3.3. MS Conditions

MS analysis was conducted in both positive and negative ionization modes to maximize the detection of diverse chemical constituents. The ESI source parameters were optimized as follows: spray voltage: 3.0 kV (positive mode) and 2.8 kV (negative mode); sheath gas flow rate: 30 arbitrary units (arb); auxiliary gas flow rate: 10 arb; capillary temperature: 320°C; heater temperature: 350°C; S‐lens RF level: 50. Full‐scan mass spectra were acquired in the range of m/z 100–1500 with a resolving power of 35,000. For MS/MS analysis, PRM was employed with a resolving power of 17,500. The normalized collision energy was set at 30 eV to ensure efficient fragmentation of precursor ions. Nitrogen (purity ≥ 99.999%) was used as the collision gas.

#### 3.3.4. Data Acquisition and Processing

Data acquisition and processing were performed using Xcalibur software (Version 4.2, Thermo Fisher Scientific) and Compound Discoverer (Version 3.0, Thermo Fisher Scientific). The raw data were processed to extract accurate mass measurements, retention times, and fragmentation patterns. Compound identification was achieved by comparing the experimental data with reference standards, literature reports, in‐house databases, and public databases (e.g., PubChem, ChemSpider). The mass accuracy threshold was set at ±5 ppm to ensure reliable identification of chemical constituents.

#### 3.3.5. Optimization of the UHPLC‐QE‐‐Orbitrap‐MS Condition

To achieve optimal chromatographic separation and detection of *C. japonicum* components, we systematically optimized both UHPLC and MS parameters through single‐factor experiments. The UHPLC conditions were carefully evaluated, including mobile phase composition (comparing acetonitrile/water and methanol/water systems with 0.05%–0.2% formic or acetic acid additives), column selection (HYPERSIL GOLD C18, 100 × 2.1 mm, 1.9 μm and Waters ACQUITY BEH C18, 100 × 2.1 mm, 1.7 μm), flow rate (0.2, 0.3, and 0.4 mL/min), column temperature (25°C, 30°C, 35°C, 40°C), and gradient elution program, and the MS parameters, including sheath and auxiliary gas flow rates, capillary resolution, and collision energy et al. Under the optimized conditions of UHPLC‐QE‐ Orbitrap‐MS, the chemical components in the *C. japonicum* exhibited good separation and fragment ion characteristics.

## 4. Discussion

UHPLC‐QE‐Orbitrap‐MS offers high resolution, superior separation performance, and enhanced sensitivity, making it highly effective for rapidly identifying chemical constituents within complex samples [28]. PRM, as a mass spectrometric analytical approach, employs high‐energy collision‐induced dissociation (HCD) fragmentation technology, enabling the collection of MS/MS spectra with elevated resolution and precise mass measurements using the Orbitrap analyzer [19].

The comprehensive chemical profiling of *C. japonicum* using UHPLC‐QE‐Orbitrap‐MS coupled with PRM identified 94 compounds, with organic acids and flavonoids emerging as the dominant classes. Among the identified constituents, chlorogenic acid and caffeic acid are the key active components responsible for the hemostatic effects of *C. japonicum* [34]. Chlorogenic acid can generate caffeic acid through hydrolysis, thereby exerting its hemostatic activity [35]. Additionally, components such as salicylic acid, quinic acid, and p‐coumaric acid, which exhibit potent anti‐inflammatory properties [36], may contribute to the anti‐inflammatory pharmacological effects of *C. japonicum*. Meanwhile, malic acid exhibits significant antibacterial activity [37, 38]. This property provides a crucial material basis for the antimicrobial effects of this herb.

The findings of this study lay an experimental foundation for systematically elucidating the chemical basis of the hemostatic and antioxidant efficacy of *C. japonicum*. Subsequent research could employ network pharmacology approaches to decipher the multitarget mechanisms of these components and utilize pharmacokinetic studies to clarify the absorption, metabolism, and pharmacodynamic patterns of key constituents such as chlorogenic acid and caffeic acid, thereby comprehensively validating the scientific value of its bioactive substances.

## 5. Conclusions

In this study, we employed the strategy of UHPLC‐QE‐Orbitrap‐MS combined with PRM to identify and characterize *C. japonicum*. Using this strategy, a total of 94 compounds were consistently and provisionally identified in *C. japonicum*. This finding significantly broadens the range of known chemical constituents of *C. japonicum*, thereby aiding in the comprehension of its active components and enhancing the quality control measures.

## Funding

The authors received no financial support for the research, authorship, and/or publication of this article.

## Conflicts of Interest

The authors declare no conflicts of interest.

## Data Availability

Data will be made available on request.
